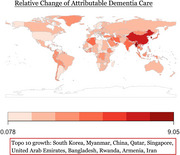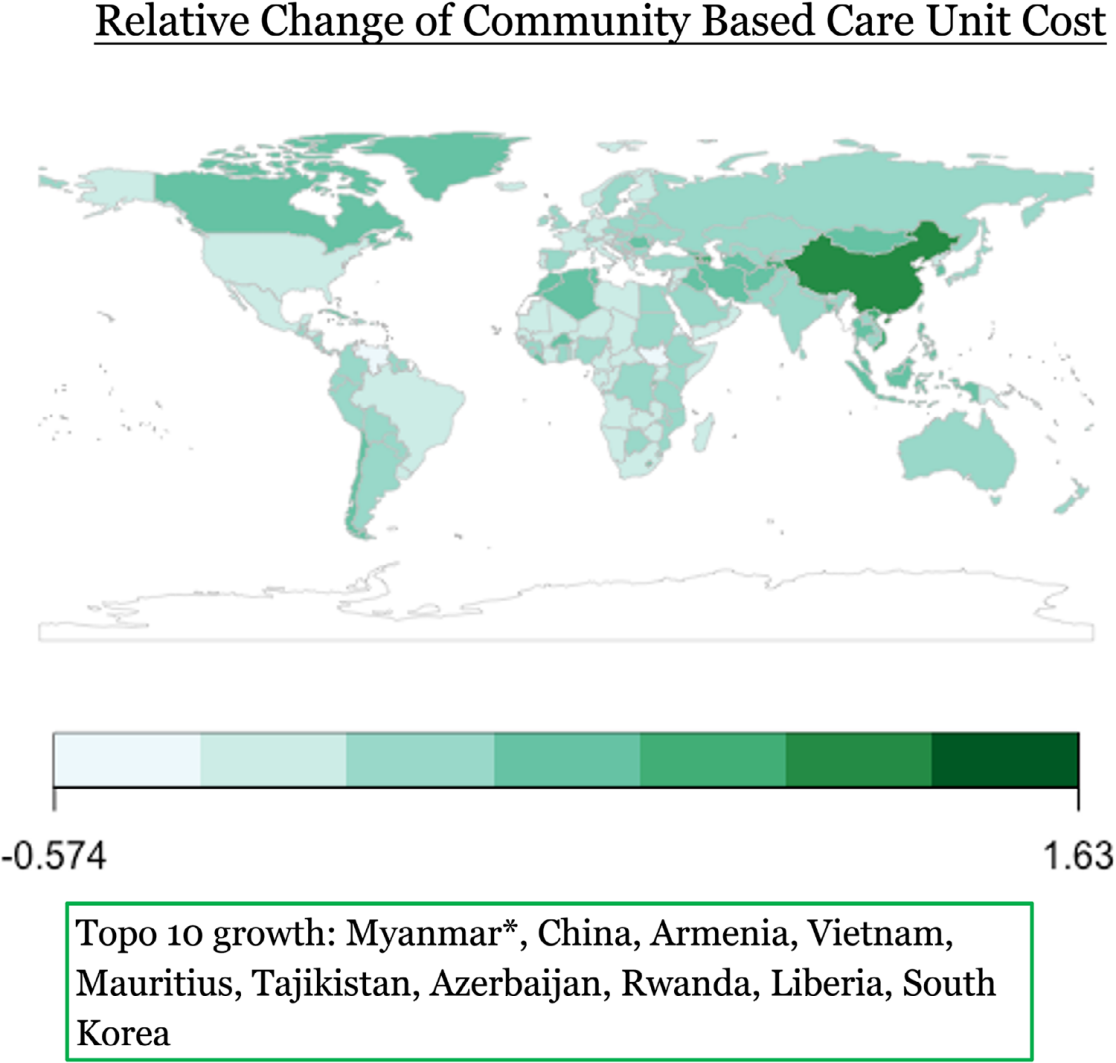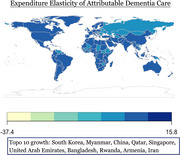# Tracking the Economic Burden of Dementia: Changes in Care Costs and Spending Elasticity Relative to GDP and Per Capita Health Expenditure

**DOI:** 10.1002/alz70860_106645

**Published:** 2025-12-23

**Authors:** YUE DONG, Sasmitha L Rajesh, Paul W Scott, Jonna M Morris

**Affiliations:** ^1^ University of Pittsburgh, Pittsburgh, PA, USA

## Abstract

**Background:**

By 2030, over 1 billion people globally will be over 65. Dementia spending, now $263 billion, is projected to reach $1.6 trillion by 2050, highlighting the need to track spending changes relative to GDP for healthcare policy planning.

**Method:**

Global dementia spending data were sourced from Institute for Health Metrics and Evaluation, GDP from the World Bank, and per capita health expenditure from World Health Organization. We used direct computation to analyze the relative dementia‐related change of community‐based care rate (CBC), facility‐based care rate (NHBC), Community based care unit cost (CBCUC), Nursing home‐based care unit cost (NHBCUC), and attributable dementia spending (ADP) from 2000 to 2019 through 195 countries. Relative change was calculated by the 2000–2019 value difference over the 2000 value. Expenditure elasticity of CBCUC, NHBCUC, and ADP was determined by dividing relative change by relative GDP growth for each country. Dementia expenditure relative change ratio (DERCR) was indicated using a ratio of relative change in ADP over relative change of capita health expenditures from 2010 to 2019 through 70 countries. Regression analysis was conducted to examine the year‐by‐year trajectory of dementia care costs for country of interest.

**Result:**

Descriptive measures will be visualized in global heatmaps. Myanmar had the largest dementia CBC increase (75.0%), Venezuela the greatest decrease (‐33.3%). Equatorial Guinea led in dementia NHBC rise (63.2%). Syria saw the highest CBCUC expenditure elasticity rise (518.9%), Brunei the largest drop (‐36.0%). Japan had the highest NHBCUC (266.6) and ADP (132.0) elasticity increases. Kyrgyzstan (<0.00%) had the lowest DERCR and Mongolia the highest.

**Conclusion:**

Drastic relative changes of global dementia care rate and cost, either increasing or decreasing, were observed for certain countries, with comparison to themselves twenty years ago. Syria and Japan are the two countries whose spending on dementia care is by proportion growing faster than the country's overall economic growth. Future steps will be made to identify growth pattern by each country.